# Biomarkers of Whole-Grain and Cereal-Fiber Intake in Human Studies: A Systematic Review of the Available Evidence and Perspectives

**DOI:** 10.3390/nu11122994

**Published:** 2019-12-06

**Authors:** Mohamad Jawhara, Signe Bek Sørensen, Berit Lilienthal Heitmann, Vibeke Andersen

**Affiliations:** 1 Focused research unit for Molecular Diagnostic and Clinical Research, IRS-Center Sonderjylland, Hospital of Southern Jutland, 6200 Aabenraa, Denmark; Signe.Bek.Sorensen@rsyd.dk (S.B.S.); va@rsyd.dk (V.A.); 2 Institute of Regional Health Research, University of Southern Denmark, 5230 Odense, Denmark; 3 Institute of Molecular Medicine, University of Southern Denmark, 5230 Odense, Denmark; 4 Department of Surgery, Hospital of Southern Jutland, 6200 Aabenraa, Denmark; 5 Research Unit for Dietary Studies, the Parker Institute, Bispebjerg and Frederiksberg, 2000 Frederiksberg, Denmark; Berit.Lilienthal.Heitmann@regionh.dk; 6 Section for General Medicine, Department of Public Health, University of Copenhagen, 2100 Copenhagen, Denmark; 7 The Boden Institute of Obesity, Nutrition, Exercise & Eating Disorders, The University of Sydney, Sydney, NSW 2006, Australia

**Keywords:** whole grains, cereal fibers, rye, wheat, oat, barley, benzoxazinoid, avenacosides, biomarker, alkylresorcinol

## Abstract

High whole-grain consumption is related to better health outcomes. The specific physiological effect of these compounds is still unrevealed, partly because the accurate estimation of the intake of whole grains from dietary assessments is difficult and prone to bias, due to the complexity of the estimation of the intake by the consumer. A biomarker of whole-grain intake and type of whole-grain intake would be useful for quantifying the exposure to whole-grain intake. In this review, we aim to review the evidence on the potential biomarkers for whole-grain intake in the literature. We conducted a systematic search in Medline, Embase, Web of Science, and the Cochrane database. In total, 39 papers met the inclusion criteria following the PRISMA guidelines and were included. The relative validity, responsiveness, and reproducibility of these markers were assessed for short-, medium-, and long-term exposure as important criteria for the potential use of these biomarkers from a clinical and research perspective. We found three major groups of biomarkers: (1) alkylresorcinol, as well as its homologs and metabolites, assessed in plasma, adipose tissue biopsies, erythrocyte membranes, and urine; (2) avenacosides, assessed in urine samples; and (3) benzoxazinoid-derived phenylacetamide sulfates, assessed in blood and urine samples. The reviewed biomarkers may be used for improved assessment of associations between whole-grain intake and health outcomes.

## 1. Introduction

Whole grains (WGs) are a rich origin of dietary fibers and numerous bioactive compounds. Each one of these compounds has various physiological functions [1]. Recent epidemiological studies suggest that the intake of WG components seems to be associated with a lower risk of various chronic lifestyle-associated diseases, particularly cancer, type 2 diabetes, obesity, and cardiovascular diseases [2,3,4,5,6], as well as better health and treatment outcomes in some inflammation-related chronic diseases [7,8], and they contribute to the human–microbe symbiosis [9,10]. Furthermore, a correlation between WGs and a greater nutrient intake and improved quality of diet was reported [11,12]. WGs are defined as “consisting of the intact, ground, cracked, or flaked caryopsis of the grain whose principal anatomical components, the starchy endosperm, germ, and bran, are present in the same relative proportions as they exist in the intact grain” [13]. Many countries promote WG consumption in their dietary guidelines [14]. The Danish official dietary guidelines recommend citizens to prioritize WG components in their diets [15]. Children over 10 years and adults are recommended to eat at least 75 grams of WG each day [16].

However, the precise mechanism of the positive physiological effects offered by WG remains unresolved [1]. The weak accuracy of assessing habitual diet intake is a common obstacle in nutritional data [17]. These data rely on self-reported dietary assessment methods that are often subject to recall bias and prone to random and systematic measurement errors [18,19]. Intake of WG may be captured with some accuracy by methods like diet history interviews specifically focusing on their intake, using the double portion method or food frequency questionnaire (FFQ) inventories designed to particularly capture WG in the diet [20,21]. However, many of the common FFQ inventories, as well as the diet record or diet recall methods, may not capture WGs in foods because they do not provide specific information on the particular product consumed of a given food or food group [22]. The use of a biomarker has the potential to measure the intake of a given nutrient objectively and with less variation, which may lead to a strengthening of the correlations between WG intake and the reduced risk of certain diseases [23,24]. A biomarker was defined by the International Program on Chemical Safety, led by the World Health Organization (WHO) and in collaboration with the United Nations and the International Labor Organization as “any substance, structure, or process that can be measured in the body or its products, can influence or predict the incidence of outcome or disease, and can be classified into markers of exposure, effect, and susceptibility” [25].

Different biomarkers of WG intake were assessed and reported during the last few decades. The evidence for their validity is difficult to synthesize because of the multitude of biomarkers and different study approaches, which makes it challenging to get a broad overview of this topic. We, therefore, performed a systematic review of results from the published literature on the validity of the biomarkers of WGs and cereal fibers that were reported in human studies in order to assess their potential use from a clinical and research perspective.

## 2. Materials and Methods

### 2.1. Search Methods

The research question was defined using the Population, Intervention, Comparator outcome, and study design criteria (PICO) [26], as presented in Table 1. We conducted a systematic search in Medline, Embase, Web of Science, and Cochrane databases for papers assessing the relative validity, responsiveness, and reproducibility of biomarkers of WG and cereal-fiber intake in humans. The used search terms are shown in Table S1 (Supplementary Materials). The cut-off date of 20 September 2019 was used with an initial limit date applied to 1975. The search was restricted to human studies, with no restrictions on the age range, gender, ethnicity or health status of the participants. The search terms were designed to limit the search to papers that provided information on biomarkers for WG intake. The search was, however, extended to articles assessing biomarkers for dietary-fiber intake, as these studies may include evidence on WG biomarkers. Relevant articles were also manually identified using the reference lists of the identified studies.

### 2.2. Selection Criteria

We included published, peer-reviewed randomized controlled cross-over and parallel studies, case–control studies, cohorts, and cross-sectional studies that evaluated or validated biomarkers of WG consumption in humans. Case reports, conference abstracts, systematic reviews, and papers in other languages than English were excluded.

### 2.3. Data Collection and Analysis

Two reviewers (M.J. and S.B.S.) screened the papers following the Preferred Reporting Items for Systematic Review and Meta-Analysis (PRISMA) guidelines [27]; any discrepancies were resolved by mutual consensus, and, when necessary, a third reviewer was added (V.A.). We used Cochrane’s online systematic review software “Covidence” in this process [28]. The variables of interest are listed in the included tables (Tables 2–4, and Table S2, Supplementary Materials). Briefly, we collected descriptive variables such as the study design, country and year of publication, and patient characteristics (age, number, sex, and comorbidities), exposure variables such as the type of the targeted fibers, method of report of the exposure, recall period if a questionnaire was used, outcome variables such as the examined potential biomarkers, used biological material, and the results.

### 2.4. Data Analysis

In order to evaluate the capability of the evaluated biomarkers, various statistical methods and related coefficients were used when assessing the association between WG intake and biomarkers in the included papers. In this review, we used the guide of Evans in the interpretation of both Pearson’s and Spearman’s correlations, as it provides a more detailed classification compared to Cohen’s guide [29,30]. Briefly, a statistically significant *r* less than 0.20 is considered very weak, 0.20 to 0.39 is considered weak, 0.40 to 0.59 is considered moderate, 0.60 to 0.79 is considered strong, and 0.80 or greater is considered a very strong correlation. We took into account the confidence interval and the *p*-value in the interpretation of the results, as these classifications referred to linear associations. In the assessment of the reported intraclass correlation coefficient (ICC), values less than 0.5 were considered poor, values between 0.5 and 0.75 were considered moderate, values between 0.75 and 0.9 were considered good, and values greater than 0.90 were considered as having excellent reliability, based on the 95% confidence interval (CI) [31]. We reported the confidence interval (CI), as well as the standard deviation (SD) or the standard error (SE), of the mean in studies when these values were reported.

Due to heterogeneity in study design, measurement, and analysis methods in different studies, the Cochrane risk of bias checklists assessment was not considered suitable for use in the present review. Instead, we used a scale based on our methodological and clinical knowledge in the field (Table S2, Supplementary Materials). The protocol of this systematic review was registered in the International prospective register of systematic reviews (PROSPERO) under CRD42019137708.

## 3. Results

### 3.1. Overview of the Studies Included

**Figure 1:**
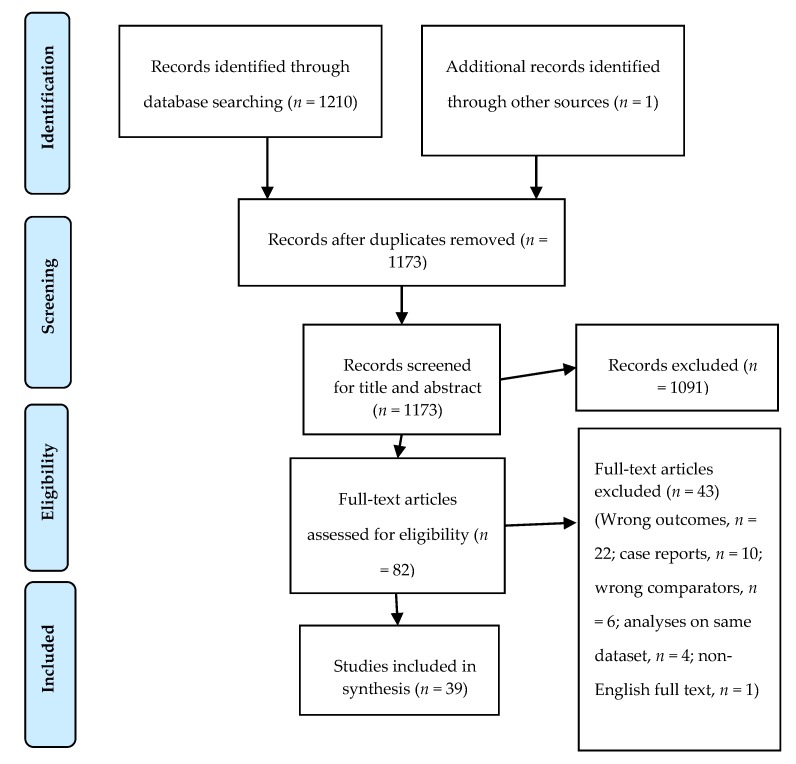
The Preferred Reporting Items for Systematic Review and Meta-Analysis (PRISMA) flow chart of database literature search and study selection [28].

We included 39 studies that met the inclusion criteria for this review (Figure 1). These studies involved 7002 participants; of them, 96 were included in more than one study [32,33,34,35]. The health status of these participants was reported in 32 studies; seven of these included 914 free-living subjects (Table 1). Seventeen studies evaluated alkylresorcinols (AR) as a biomarker for WG intake in plasma (P-AR) [34,35,36,37,38,39,40,41,42,43,44,45,46,47,48,49,50], one study in erythrocyte membranes [51], and three studies in adipose tissue [43,47,50]. AR metabolites were evaluated in urine in 12 studies [32,33,34,39,42,52,53,54,55,56,57,58], and in plasma in five studies [52,56,59,60,61]; one study evaluated plasma benzoxazinoid compounds (N-(2-hydroxyphenyl) acetamide (HPAA) and hydroxy-N-(2-hydroxyphenyl) acetamide (HHPAA sulfate)) as biomarkers of whole-grain rye intake (WGR) [62]; one study evaluated urinary avenacosides as a biomarker for oat intake [63]. Seven studies applied a non-targeted metabolomic approach [64,65,66,67,68,69,70]. This review included nine cross-over [36,37,38,39,40,42,44,64,65] and five parallel randomized controlled studies [41,43,45,62,66], three case–control studies [46,56,60], 16 cohort studies [32,33,34,35,47,51,52,54,55,57,58,59,61,63,67,68], and six cross-sectional studies [48,49,50,53,69,70]. Fourteen studies were conducted in Sweden [32,33,35,38,39,44,47,50,55,57,60,65,68,70], 10 in Finland [34,40,43,51,52,59,61,62,64,66], seven in the United States of America (USA) [42,49,53,54,58,63,67], two in Denmark [37,48], two in the United Kingdom (UK) [36,45], two as multicenter European studies [41,46], one in Spain [69], and one in Latvia [56]. The main characteristics, the targeted biomarkers, and main findings of the included studies are presented in Table 2. Studies applying a non-targeted metabolomic approach are described in Table 3, and the main results are presented in Table 4.

### 3.2. Quality Assessment and Assessment of the Risk of Bias in the Included Studies

Table S2 (Supplementary Materials) shows the quality assessment and the assessment of the risk of bias of the included studies. Of 39 studies, 32 studies reported their inclusion and exclusion criteria [32,33,34,35,36,37,40,41,42,43,44,45,46,47,49,52,53,54,55,56,58,59,60,61,62,63,64,65,66,67,69,70], whereas 27 studies reported the health status of the included subjects [34,35,36,37,39,40,41,42,43,44,45,46,49,52,53,56,58,59,60,61,62,63,64,65,66,67,70]. Only one study reported the blinding of the assessors [65], and nine studies reported that the used questionnaires was validated specifically for the study [32,33,34,45,46,52,57,59,69]. Twenty-three studies reported a recall period for the used questionnaire of less than 10 days [32,33,34,35,36,37,38,39,41,42,43,44,45,49,52,55,56,59,60,64,65,66,70], and 19 studies used the questionnaire data to assess a period over one week [36,37,38,40,41,43,44,45,47,48,49,50,51,53,54,64,65,66,69]. Nineteen of 27 studies assessing biomarkers in plasma used fasting samples [34,36,37,38,39,42,43,44,45,47,50,51,52,56,59,61,62,65,66]. Of studies assessing biomarkers in urine, nine of 18 studies used 24-h urine [32,39,42,55,58,64,67,68,70], and three used spot urine [33,54,69].

### 3.3. Reported Biomarkers

#### 3.3.1. Alkylresorcinols in Plasma

**Figure 2:**
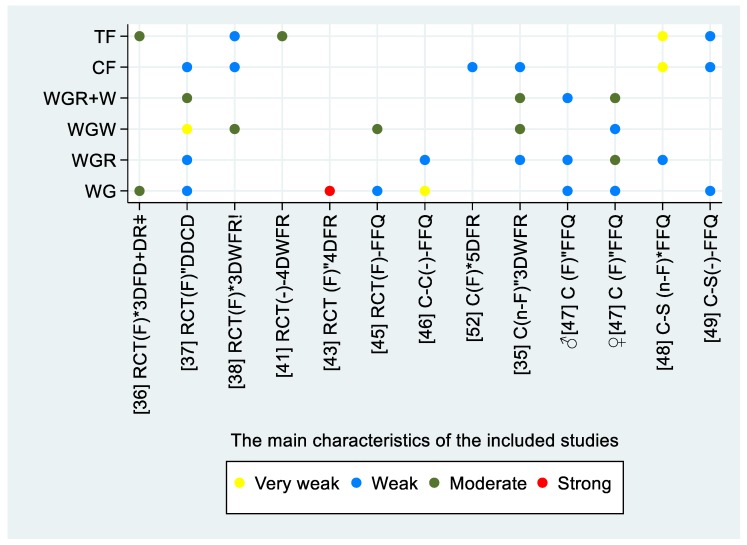
The reported correlations between the intake of whole grains and alkylresorcinol concentration in plasma. The figure includes information on study design, sampling condition, sampling time, and dietary assessment method on the horizontal axis. The vertical axis represents the type of the exposure. Abbreviations: study design—randomised controlled trial, RCT; case–control, CC; cohort, C; cross-sectional, C-S; sampling condition; fasting, (F); non-fasting, (n-F); sampling time—* within 24 h since last intervention/intake; “ later than 24 h after; dietary assessment method—three-day food diaries, 3DFR; daily dietary compliance diaries, DDCD; three-day weighted food records, 3DWFR; four-day food intake records, 4DFR; food frequency questionnaire, FFQ; whole grain, WG; whole-grain rye, WGR; whole-grain wheat, WGW; cereal fiber, CF; total fiber, TF; correlation—very weak, *r* < 0.20; weak, 0.20 ≤ *r* ≤ 0.39; moderate, 0.40 ≤ *r* ≤ 0.59; strong, 0.60 ≤ *r* ≤ 0.79; ǂ analyzed separately; ! pooled data.

Seventeen studies that assessed alkylresorcinol in plasma (P-AR) were included in this review [34,35,36,37,38,39,40,41,42,43,44,45,46,47,48,49,50]. Thirteen studies reported the relative validity of P-AR and reported the correlation between P-AR and the exposure to different WGs [34,35,36,37,38,40,41,43,45,46,47,48,49]. For the correlation between P-AR and whole-grain rye (WGR), four studies reported a weak correlation [35,37,46,48]. One study reported a weak correlation among the men, and a moderate correlation among the women [47]. Three studies reported a moderate correlation between P-AR and whole-grain wheat (WGW) [35,38,45], one study reported a very weak correlation [37], and one study reported a weak correlation among women; no correlation was reported among men [47]. Three studies reported a moderate correlation between P-AR and whole-grain rye and wheat (WGR + WGW) [35,37]. Wu et al. reported a weak correlation among men and a moderate correlation among women [47]. Five studies reported a weak correlation with cereal fibers [34,35,37,38,49], while one study reported a very weak correlation [48]. Two studies reported a moderate correlation to total fibers [36,41], and two other studies reported a weak correlation [38,49], while one reported a very weak correlation [48]. The correlation between P-AR and total WG intake was reported to be weak in four studies [37,45,47,49], very weak in one [46], moderate in one [36], and strong in one study [43]. The study design, sampling condition and time, the used dietary assessment method, and the reported correlations to the intake are illustrated in Figure 2. Correlation coefficients, CI intervals and *p*-values are presented in Table 2 when available in the included papers.

The sensitivity of AR to the WG dose change is poor, both in short- and long-term exposure. McKeown et al. reported in their study that the short-term dose response of the mean of P-AR on the WGW was significantly higher after two one-week interventions of three and six daily servings of WGW than at the wash-out (>/3.1-fold higher). No significant dose–response difference was found between the two interventions [42]. In another study, Ross et al. assessed the long-term dose response of P-AR in three intervention groups of WG in a parallel randomized controlled trial with three intervention groups of low, medium, and high intake of WG. After 16 weeks, a significant difference in P-AR was shown between the group with the low WG intake and the other groups. No significant difference in P-AR was demonstrated between the medium and the high WG intake groups [45]. A study from Andersson et al. showed a strong correlation between P-AR homologs C17:0 and rye intake, and a moderate correlation between C21:0 and the wheat intake. The authors reported a moderate correlation between the C17:0/C21:0 ratio and the WGR and a moderate inverse correlation with the WGW intake [35]. A similar good specificity of the C17:0/C21:0 ratio with the rye/wheat intake was reported in three other controlled intervention conditions [38,39,40]. No correlation was observed between AR homologs and barley, oat, corn, or rice intake [35], while a weaker correlation with the WG intake was observed in subjects with higher consumption of non-A- containing grains [36]. Landberg et al. reported good short-term reproducibility (six weeks) of plasma AR under intervention conditions where the intake of WGR was high and kept constant [44]. The reproducibility of plasma AR was poor (ICC = 0.47; 95% CI: (0.27, 0.67)) over a 2–3-month period among free-living subjects [35].

#### 3.3.2. Alkylresorcinol in Adipose Tissue Biopsies

Three studies evaluated the correlation between WG intake and AR in adipose tissue biopsies. In a cross-sectional study design, Jansson et al. reported a moderate correlation between WG bread and total AR in adipose tissue [50]. In a randomized cross-over parallel study of WG and refined-grain (RF) interventions over 12 weeks, Wu et al. reported a strong correlation between WG intake and both P-AR (*r* = 0.60–0.72, *p* < 0.05) and AR in adipose tissue (*r* = 0.60–0.84, *p* < 0.05), and a higher P-AR and AR in adipose tissue in the WG than the RF intervention [43]. In a retrospective cohort study design, Wu et al. evaluated AR in adipose tissue biopsies as a biomarker of long-term WG wheat and rye intake in women (over one, seven, and 17 years) and in men (over one, two, and 14 years), and they found weak correlations with WG, WGR, WGW, and WGR + WGW intake in both genders except for a moderate correlation with WGR in women over one, seven, and 17 years [47].

#### 3.3.3. Alkylresorcinol in Erythrocyte Membrane

Only one study addressed AR in the human erythrocyte membrane (EM) as a biomarker of WGW and WGR intake. In a parallel controlled study design, Linko et al. demonstrated that AR is incorporated and can be measured in the human EM in vivo. They also demonstrated a good symmetric progression of AR in plasma and EM in response to the WGR, WGW, and WG barley intake. No AR was detected in a subject gluten-free diet, nor was it detected in EM or plasma [51]. The composition of AR homologs differed between plasma and EM samples. The average percentage of C17:0 was significantly higher in plasma (13% CI (6, 16)) compared to the average percentage in EM (5% CI (3, 9)) (*p* < 0.001). In contrast, the average percentage of C25:0 was higher in EM (5% CI (4, 9)) compared to plasma (12% CI (10, 13)) (*p* < 0.001) [51].

#### 3.3.4. Alkylresorcinol Metabolites in Plasma

Five studies assessed the plasma levels of the two AR metabolites, 3,5-dihydroxybenozoic acid (DHBA) and the 3-(3,5-dihydroxyphenyl)-1-propanoic acid (DHPPA), as potential biomarkers for WG intake [34,56,59,60,61]. Drake et al. reported in a case–control study design a very weak correlation between DHBA, DHPPA, and DHBA + DHPPA with cereal fibers, a week correlation with total WG, high-fiber bread, and total fiber intake, and a very weak inverse correlation with low-fiber bread in non-fasting samples [60]. Another study found a moderate correlation between both metabolites and their sum and total cereal fiber in the fasting samples [59], while no significant correlations were detected between these metabolites and vegetable, berry, or fruit fiber intake in another study [59]. Soderholm et al. reported good responsiveness of both metabolites in response to the range of WGR intake. After a washout period and a single WGR dose intake, the plasma concentration of DHBA and DHPPA raised simultaneously and reached a c-max after six hours (DHBA-t_max_ = 6.1 ± 0.5 h and DHPPA-t_max_ = 6.4 ± 0.7 h). The concentration of each metabolite at 25 h was slightly but significantly higher than at baseline. The t_1/2_ of DHPPA was longer compared to DHBA (t_1/2_-DHPPA = 16.3 ± 1.8 h, t_1/2_-DHBA = 10.1 ± 0.8 h) [61].

#### 3.3.5. Alkylresorcinol Metabolites in Urine

Three studies assessed the relative validity of AR metabolites in spot urine; all studies addressed validity among free-living subjects. Four studies addressed AR metabolites in 12–36-h urine. DHPPA and DHBA are the major relative components of AR metabolites [32,58]. The proportion of the individual metabolite in the total AR metabolite excretion in 24-h urine was 42% for DHPPA, 33% for DHBA, 13% for DHCA, 9% for DHBA-glycine, and 2% for DHPPTA [32]. Landberg et al. estimated the four-day intake of WG. It correlated with the mean creatinine-adjusted AR metabolite concentrations from four spot-urine samples collected during the same period (days one, two, 13, and 14): DHBA (*r* = 0.49, *p* < 0.05), DHPPA (*r* = 0.38, *p* < 0.05), DHCA (*r* = 0.49, *p* < 0.05), DHBA-glycine (*r* = 0.42, *p* < 0.05), and DHPPTA (non-significant). Generally moderate to very strong correlations between metabolites were reported (except a very weak correlation between DHPPTA and DHPPA) [33]. In another study where DHBA, DHPPA, and DHBA + DHPPA were assessed as long-term biomarkers (2–3 years), very weak to weak correlations were reported with WG, cereal fiber, and total fiber intake, as well as a poor reproducibility of these metabolites between the two time points [54]. Marklund et al. compared the relative validity of DHBA, DHPPA, and DHBA + DHPPA between spot urine and 24-h urine. In spot urine, they reported a moderate correlation between DHPPA and WG, WGR, and WGR + WGW. The association with WGW was not significant. Similar results were reported for DHBA, except that, here, the authors found a strong correlation with WGR + WGW. In 24-h urine, slightly better correlations were reported for DHBA + DHPPA, and similar correlations were reported for the two other metabolites [55]. DHBA, DHPPA, and DHBA + DPPPA did not correlate with either the oat, barley, or rice intake. The authors reported a poor reproducibility of the concentration of DHBA and DHPPA in 24-h urine (ICC = 0.46–0.51), and a poor reproducibility in spot urine between two occasions three months apart, even when no difference in WG consumption between two occasions was observed [55].

Guyman et al. reported that the excretion of DHPPA in 12-h urine was 44% higher in whole-grain wheat and rye consumers than non-consumers after adjusting for body mass index (BMI), and energy and fiber intake [53]. Aubertin-Leheundre et al. evaluated the relative validity of the concentration of DHBA and DHPPA in 72-h urine as biomarkers for rye and cereal-fiber intake, based on a five-day food record that was initiated two days before the specimen collection. They reported a weak correlation between cereal fibers and DHBA and a moderate one with DHPPA. Generally, DHBA and DHPPA correlated modestly with P-AR and all AR homologs [34]. In a similar study set-up, Aubertin-Leuheundre observed a strong correlation between WGR intake and DHBA, and a moderate correlation with DHPPA. The authors observed a slightly weaker correlations between the total fiber intake and DHBA (*r* = 0.443 *p* < 0.05) and DHPPA (*r* = 0.390 *p* < 0.05) [52]. Recently, Wierzbicka et al. reported a strong correlation between the WGR intake and DHCA in 24-h urine samples on two occasions 2–3 months apart. A moderate correlation was observed for WGR with DHBA and DHPPTA (5-(3,5-dihydroxyphenyl) pentanoic acid), and for WGW intake with DHBA, DHPPA, and DHCA (3,5dihydroxycinnamic acid) on the first occasion. No correlations were observed between WGW and these metabolites on the second occasion, which was explained by a lower and less stable WGW intake on the second occasion. The intake of WG oats, barley and maize, did not correlate with the AR metabolite concentrations. DHCA-amide (3,5-dihydroxycinnamic acid amide) did not correlate with AR intake even if it was the metabolite with the highest urinary excretion, and the authors suggested that DHCA-amide may have a precursor other than AR [32]. McKeown et al. reported a good responsiveness of 24-h urine DHBA and DHPPA to the WG intake in an RCT study design. DHBA, DHPPA, and DHBA + DHPPA excreted in urine after WGW intake were higher compared with washout and higher when the WG intake increased from three to six servings daily [42]. Similar findings were reported in other studies [39,58]. The pharmacokinetic parameters indicate that the half-life (t_1/2_) of DHBA is slightly longer than DHPPA in urine (15.9 h vs. 14.8 h) [58].

#### 3.3.6. Avenacosides

Wang et al. investigated the metabolism and pharmacokinetics of avenacosides as a biomarker of oat intake in humans, and it was reported to be absent in urine after the washout period, and present two hours after the ingestion of a single dose of WG oat. Only a trace of these metabolites was present 36 h after the exposure [63].

#### 3.3.7. Benzoxazinoid-Derived Phenylacetamide Sulfates

One study assessed the plasma profile of the double-hexose-conjugate of 2,4-dihyxdoxy 1,4-benzoxazin-3one (DIBOA) in subjects after consuming WGR, WGW, and refined grain bread [62]. Fasting plasma samples were collected at four time points during 24 h after the exposure and were analyzed by an LC–quadrupole time-of-flight (QTOF)-MS approach [62]. Hydroxy-*N*-(2-hydroxyphenyl) acetamide (HHPAA) and *N*-(2hydroxyphenyl) acetamide (HPAA) appeared in the plasma 60 min after the intake of WGR bread and reached their maximal concentration at 120 min and 60 min, respectively. Both metabolites were absent after 24 h of exposure. HHPPA and HPAA were also detected in other studies assessing plasma and urine metabolites after WG exposure by a non-targeted metabolomic approach [64,67,69,70] (Table 3).

#### 3.3.8. Untargeted Metabolomics Studies

Seven metabolomic studies with an untargeted approach met the inclusion criteria of this review [64,65,66,67,68,69,70]. The majority of them aimed to elucidate metabolites associated with WG intake. Two of these studies explored metabolites in fasting plasma [65,66], two in 24-h urine [64,70], one in 24-h urine collected at six different time-points [67], one in two-day 24-h urine [68], and one in spot urine [69]. Table 3 shows the main characteristics of these studies. The reported databases, known metabolites, and the main results are listed in Table 4.

## 4. Discussion

In this study, we systematically reviewed the available potential biomarkers for WG intake in humans. We found three major groups of biomarkers: (1) AR, as well as its homologs and metabolites, assessed in plasma, adipose tissue biopsies, erythrocyte membranes, and urine; (2) avenacosides, assessed in urine samples; and (3) benzoxazinoid-derived phenylacetamide sulfates, assessed in blood and urine samples.

AR, its homologs, and metabolites were the predominant group of the assessed biomarkers for WG intake. They showed good responsiveness and generally a moderate to strong short-term relative validity for WGR and WGW intake. However, some studies reported weaker correlations when assessing the relative validity of AR and its metabolites as biomarkers of the WG, WGR, and WGW intake. Different factors may contribute to these differences, such as the design, the methods, and the set-up of the studies. Stronger correlations were generally observed in RCTs compared to studies with a cross-sectional design. Yet, one study with an RCT design reported a non-significant difference in the mean concentration of P-AR between groups with high and low intake of WG [37]. Secondly, the included subjects across the assessed studies had different health status and comorbidities. The absorption, metabolism, and excretion processes of these compounds may vary depending on the clinical status of the involved organs and tissues [23,71,72,73], and may, thus, contribute to the discrepant results. Thirdly, the concentration of AR in WGR and WGW was previously found to vary widely between 360 and 3200 μg/g and 317 and 1429 μg/g, respectively [74,75,76]. This may contribute to explaining that the concentration of AR, its homologs, and metabolites in biological samples varied with the type of consumed WG in the different studies. Lastly, AR and its homologs are present in high concentrations in WGR and WGW, and in very low concentrations in maize, peas, triticale, and barley grains; they are absent in oat and rice [77,78]. The intake of oat, spelt, maize, millet, rice, and sorghum and other non-AR-containing WG contributes to the total WG intake, but not to the concentrations of AR, its homologs, or its metabolites [36,43,47,55]. In an included WG intervention study, the 12 subjects with the relatively lowest concentrations of plasma AR consumed more WG oats and less WG wheat, rye, barley, rice, and corn, compared to the rest of the subjects [36]. Thus, the concentration of AR, its homologs, and metabolites can be misleading in populations and subjects where WGR and WGW are not the primary content of WG. This review includes studies on different ethnical and geographical populations, which may also argue for the varying results.

Avenacosides were suggested as a marker for WG oat intake, primarily because these phytochemical steroid glycosides are uniquely produced in oats [79,80]. The evidence on whether avenacosides might serve as a biomarker for WG oat intake is still limited, and further research is needed. Perhaps the determination of the concentration of Avenacosides in urine could be complementary to the concentration of AR as biomarkers for WG intake, but further research is needed. Benzoxazinoid, primarily DIBOA, is the most abundant compound in different WG bread, and HHPAA and HPAA were identified after the intake of different forms of rye bread [62,81], and they were suggested as biomarkers for WG intake. In addition to WGR and WGW, they are richly found in maize and, thus, have the potential to be supplemental for AR and avenacosides in the assessment of the total WG intake. Studies with a targeted approach assessing the relative validity and the responsiveness of these compounds to the WG exposure are needed to reveal their validity.

Different multivariate statistical models combining different metabolites were proposed and showed good potential to predict the WG intake in humans [69,70]. These models are, however, mainly based on markers described before, which are limited with their short lifetime, in combination with other metabolites like enterolactone, glucuronide, and pyrraline [69]. These metabolites are not specific for WG, as they are also present in the endosperm, food additives, and many plant foods [82]. These limitations make these statistical models less promising to serve as good indicators for WG intake in a clinical or epidemiological context.

Studies on AR, its homologs, and metabolites generally showed a moderate short-term reproducibility, but a relatively poor reproducibility in the assessment of the medium- to long-term exposure to WG in the blood and urine [35,55,83,84]. To our knowledge, no study assessed the reproducibility of avenacosides and benzoxazinoid compounds. However, it is known that AR, avenacosides, and benzoxazinoid compounds share a short lifetime (<24 h) in the blood and urine. A poorly reproducible biomarker requires an extensive number of samples, at different time points, and it can still lead to biased judgments of the biomarker–disease correlation [85]. Thus, these compounds may serve as a good supplement to food frequency questionnaires and as qualitative markers of compliance in research. Their value as quantitative markers in the assessment of a habitual longer-term WG intake is, however, limited [62]. In this context of finding trustful markers of long-term intake, which is essential in both research and clinical work, other biological sample sites were investigated. The concentration of AR and its homologs in biopsies from adipose tissues generally showed a similar relative validity and reproducibility compared to those measured in plasma [43,47,50]. Equally important to the need for a validated and standardized analysis method, factors like the low price, high sensitivity, specificity, reliability, and especially less invasion determine whether a biomarker is good [86,87]. Compared to a simple peripheral blood drawing procedure, the tissue extraction procedure may be more invasive, expensive, time-consuming, and related to several clinical complications. These considerations make adipose tissues as a biomaterial less optimal in this context. Other attempts were made to assess AR in other, less invasive biological samples. It was demonstrated that AR is present in the erythrocyte membrane in subjects consuming WG. The evidence whether the concentration of AR in EM could serve as a biomarker for WG intake is still limited, and further research is needed [51]. To date, trustful biomarkers measuring the medium- and long-term WG intake are still missing.

There are several limitations to the results of this review that we should acknowledge. Firstly, this review included studies with different design and methods, which made a meta-analysis of the results not suitable. Nonetheless, the integration of the results of all these studies helped to cover the topic broadly. Secondly, our review did not interpret the difference in results across the countries, where the different studied populations were included. Different communities may have a different intake of the different types of WG, which could be important to take into consideration when comparing the results of these studies. Thirdly, we decided to use a generic checklist in the assessment of the quality and risk of bias of the included articles, notwithstanding the design, measurement, and analysis methods in these studies. We acknowledge that this decision might cause some design- and analysis-specific bias to be missed. Fourthly, the health status of the populations assessed in the majority of the included studies was different. Populations with one reported diagnosis may also suffer from other unreported diseases, which made these groups non-comparable.

Recently, Landberg et al. published a scoping review of biomarkers of different cereal types including whole and refined grains, pasta, rice, and pseudo-cereals [88]. To our knowledge, this work is the first systematic review that collected the results on the validity of the available biomarkers of WG intake in humans. The methods used in this review followed the PRISMA guidelines for systematic reviews, a study protocol was registered before the study start, and the contribution of different experts strengthened the quality of the reported data. Findings from this review have important implications for epidemiological and clinical research. In the future, more research might approach and reveal biomarkers with a greater reproducibility and validity in the assessment of the medium- to long-term consumption of WG. RCT and observational study designs have different advantages in nutritional research [18]. Future studies may benefit from the Consort and the Strobe guidelines that were established to improve the quality of these studies [89,90].

## 5. Conclusions

This review evaluated potential biomarkers for whole-grain intake in humans. Because biomarkers can accurately assess intake, they can be used for improved assessment of associations between whole-grain intake and health outcomes. Alkylresorcinol and their metabolites showed good responsiveness and short-term relative validity for whole-grain rye and wheat intake. They may potentially be used in research when the assessment of the short-term intake of whole-grain rye and wheat is needed. Their poor medium- to long-term reproducibility is a substantial limitation to their use in clinical settings. Furthermore, other whole grains like oats and maize would contribute to whole-grain intake and cannot be captured by these markers. Avenacosides are present in oat and were suggested as a biomarker for whole-grain oat intake. Potentially, avenacosides could serve as a supplementary marker to alkylresorcinol in the assessment of whole-grain intake, but the evidence is still limited. Benzoxazinoid derivates were proposed as potential markers for whole-grain rye and wheat intake, but, like alkylresorcinol, they are limited by their short half-lives. More research is needed to compare the relative validity and responsiveness of these derivates to alkylresorcinol. Metabolomic studies showed a potential validity when various compounds were combined in a model to assess whole grain intake. To date, biomarkers for the assessment of the medium- and long-term whole-grain intake are missing. The concentration of the revealed biomarkers may rather serve as a supplement to food frequency questionnaires and qualitative markers of compliance rather than as trustful markers of intake measure.

## Figures and Tables

**Table 1:** The description of the PICO ^1^ criteria used for this review.

**Population**	Men and women, with no restrictions on age, ethnicity, or comorbidities
**Intervention**	WG intake
**Comparator**	Not applicable
**Outcome**	Biomarkers for WG ^2^ intake
**Study Design**	Randomized controlled trials (cross-over and parallel study designs), case–control studies, cohorts, and cross-sectional studies
**Research Question**	Which biomarkers of whole-grain intake were assessed in the literature?

^1^ PICO: Population, Intervention, Comparator outcome and study design; ^2^ whole grains.

**Table 2:** The summary of the characteristics and the main results of the included studies.

Study (Author, Year, Country)	Study Design	Population (*n*) ^1^ (% ♀) ^2^ Age (years) ^3^ Health Status	Aim (A), Intervention (I), Washout (Wo) (Background Diet)	Method of the Report of the Exposure	Biomarker Biological Sample Analytical Method	Main Results
Ampatzoglou, 2015 [36], UK	RCT cross-over non-blinded	(33) (64%) 48.8 ± 1.1 (40–65) Healthy	(A) To investigate the compliance to the WG diet data with plasma alkylresorcinol (P-AR) I1: WG (WG > 80 g) 6 weeks; Wo: (-) 4 weeks I2: RG (WG < 16 g) 6 weeks (Habitual diet controlled for WG, without prebiotics or probiotics)	3-day food diaries (3DFDs) and daily records (DRs) (analyzed separately)	Alkylresorcinol (AR) Plasma (F) LC–MS	A moderate significant correlation between (1) P-AR and total WG form both from the 3DFDs (r_s_ = 0.46, *p* < 0.001) and DRs (r_s_ = 0.52, *p* < 0.001), total fiber and P-AR WG (r_s_ = 0.46, *p* < 0.001), and (2) total fiber (r_s_ =0.40, *p* < 0.001) from the 3DFDs (r_s_ from DRs not reported). P-AR in I1 (x = 161 ± 31 nmol/L) was significantly different from I2 (x = 38 ± 5 nmol/L) and from baseline (*p* < 0.001).
Biltoft-Jensen, 2016 [37], Denmark	RCT cross-over non-blinded	(750) (49%) 0.2 ± 0.6 (8–11) Without severe disorders ^4^	(A) To validate WG intake data from 2 diets reported by children, using P-AR I1: High in WG (x = 42 CI (35–49) g) 3 months I2: Low in WG (x = 35 (29, 42) g) 3 months (Lunch controlled for WG, other meals of habitual diet)	Daily dietary compliance diaries (after each meal 4–7 days/1 week)	AR Plasma (F) GC–MS	Very close WG exposure in both intervention groups. No difference in P-AR between both groups. Weak correlation between P-AR and total WG, WGR, and total cereal fibers in both groups. Very weak to weak correlation between WGW and P-AR.
Landberg, 2008 [38], Sweden	RCT cross-over non-blinded	(30) (73%) 59 ± 5 - -	(A) To study the correlation between P-AR and WG intake I1: WG 112 g/day (18 g fiber) 6 weeks Wo: (-) 6-weeks I2: RG 112 g/day (6 g fiber) 6 weeks (Habitual diet controlled for WG cereals)	3-day weighted food records (pooled) and food diaries (for compliance check)	AR Plasma (F) GC	Significant difference between P-total AR x = 202 ± 107 in I1 and x = 59 ± 57 in I2 (*p* < 0.0001), and baseline (*p* < 0.0001). Generally, the correlation between P-AR and (1) AR and WGR + WGW intake was moderate, and (2) total fiber was weak.
Landberg, 2009 [39], Sweden	RCT cross-over non-blinded	(16) (53%) 30.6 ± 10.3 - Healthy	(A) To assess the responsiveness of P-AR and the excretion of U-DHBA and U-DHPPA in 24-h urine. I1: High WG (90 g) 1 week I2: Medium WG (45 g) 1 week I3: Low WG (22.5 g) 1 week Wo: No WG (0) 4 × 1 week (Habitual diet controlled for cereal and table spread products (provided))	Daily dietary compliance diaries (after each meal)	AR Plasma (F) U-DHBA and U-DHPPA (24-h urine) GC	P-AR differed significantly between all doses for all homologs except for 17:0/21:0 (*p* < 0.05). U-DHBA, U-DHPPA, and U-DHBA+DHPPA excretion increased significantly with dose increases (*p* < 0.001) and differed between all three doses (*p* < 0.020).
Linko, 2005 [40], Finland	RCT cross-over non-blinded	(39) (100%) 59 ± 0.94 - Hypercholesterolemia and BMI of 20–33 kg/m^2^	(A) To assess the possible utility of ARs as biomarkers for WGR and WG wheat (WGW) intake. I1: High-fiber rye bread 8 weeks Wo: Habitual eating 8 weeks I2: Low-fiber wheat bread 8 weeks (Habitual diet controlled for bread products (provided))	4-day food intake records during each intervention	AR Plasma (-) Enterolactone GC–MS	The correlation between P-AR and (1) intake of rye bread was weak (*p* < 0.05), and (2) intake of wheat bread was absent. The correlation between P-enterolactone and (1) consumption of rye bread was very weak (*p* < 0.05), and (2) intake of wheat bread was absent.
Hanhineva, 2014 [62], Finland	RCT cross-over non-blinded	(12) (-) 57 ± 9 - Almost healthy ^5^	(A) Benzoxazinoid as biomarkers for WG intake I1: Rye (high WG) I2: White wheat (low WG) (Breakfast controlled for WG. Other meals: habitual diet, no alcohol)	Not used In-clinic intervention	Benzoxazinoid compounds (HPAA and HHPAA sulfate) Plasma (F) LC–QTOF-MS	HPAA and HHPAA appeared in plasma rapidly after I1 t-max HPAA = 60 min, t-max HHPAA = 120 min. HPAA and HHPAA were not detected in I2.
Wu, 2015 [43], Finland	RCT parallel non-blinded	(16) (-) - (47–65) Metabolic syndrome	(A) To evaluate the response of adipose tissue AR after a 12-week dietary WG intervention. I1: WG (12 weeks) I2: RG (12 weeks) (Habitual diet controlled for cereals)	4-day food intake records	AR Plasma (F) and adipose tissue GC–MS	After 12 weeks, AR concentrations in the plasma and adipose tissue were significantly higher in I1 than I2 (*p* < 0.05). Strong correlation between WG intake and P-AR (r^13^ = 0.60–0.72, *p* < 0.05) and adipose tissues (r = 0.60–0.84, *p* < 0.05)
Magnusdottir, 2013 [41], European multicenter	RCT parallel non-blinded	(158) (65%) 54.5 ± 8.2 (30–65) Metabolic syndrome	(A) To assess P-AR as biomarker in Nordic diet (rich in dietary fibers) I1: High fiber (WGR + barley + oat + fruits + vegetables) (>36 g/day fibers) I2: Low fiber (RG wheat) (total fibers > 16 g/day at 18 or 24 weeks) (Controlled feeding trial)	4-day weighted food records (consecutive days) with either weighted or estimated portion sizes	AR Plasma (-) GC–MS	Significant difference between I1 (P-AR = 106) and I2 (P-AR = 61) at week 12 (*p* < 0.001). The correlation between total fiber intake and P-AR was (1) very weak at week 12 in both groups independently, and moderate when pooled, and (2) weak at the endpoint in both groups independently, and moderate when pooled.
McKeown, 2016 [42], USA	RCT cross-over non-blinded	(19) (47%) 25.6 ± 5.8 (18–40) Healthy	(A) To compare the short-term, dose response of WGW on P-AR and U-AR-metabolites. I1: High in WG wheat (-) 6 days Wo: habitual diet (no WG) 2 weeks I2 (A) WG wheat (3 days) and (B) refined wheat (3 days) (Habitual diet controlled for WGW and RF)	3-day diet record	AR Plasma (F) and AR metabolites Urine (24 h) (last day I/Wo) UHPLC	Adjusted x P-AR in I1 and I2 (A) was ≥3.1-fold higher (*p* < 0.001) than Wo. No difference between x P-AR in I1 and I2 (A) x U-DHBA, DHPPA, and DHBA + DHPPA I1 and I2 (A) were different (*p* < 0.001) from Wo. The excretion of metabolites after I2 (A) was 3.7-fold greater than WO. The mean percentage increase of metabolites for 3 WG servings compared with 6 WG servings was 75%.
Ross, 2012 [45], UK	RCT parallel non-blinded	(266) (50%) - - Overweight healthy	(A) To evaluate plasma ARs in a long-term intervention in subjects with a low habitual intake of WGW. I1: WG (60 g) 16 weeks I2: WG (60 g) 7 weeks then (120 g) 8 weeks I3: Low WG diet (<30 g) 16 weeks (Habitual diet controlled for WG)	149-question semi-quantitative FFQ	AR Plasma (F) GC–MS	After 8 weeks, a significant difference in P-AR between I1 and I2 (*p* = 0.002) and the control group (*p* < 0.0001). After 16 weeks, no difference in P-AR between I1 and I2. A significant difference between I1 + I2 and I3 (*p* < 0.0001). Total P-AR was weak correlated to total WG and AR intake (*p* < 0.001) and moderate to WG wheat (*p* < 0.01)
Landberg, 2009 [44], Sweden	RCT cross-over non-blinded	(17) (0%) 73.5 ± 4.6 - Prostate cancer	(A) To investigate the effect of very high AR intakes on fasting plasma AR concentration and to assess the short-term (6 weeks) reproducibility under intervention conditions where the intake was kept constant. I1: Rye WG 6 weeks Wo: (-) 2 weeks I1: Refined wheat 6 weeks (Habitual diet controlled for cereal and table spread products (provided))	4-day weighted food records	AR Plasma (F) (8 samples/participant) GC	P-AR plasma concentration was 991 ± 794 nmol/L in I1 and 75 ± 92 nmol/L in I2. Carry-over effect in participants starting with I1 (P-AR was higher in Wo and I2) for C19:0, C21:0, C23:0, and for total AR. The AR C17:0/C21:0 ratio was higher in I1 (0.65 ± 0.24) than I2 (0.27 ± 0.22) (*p* < 0.0001). Good reproducibility of P-AR under intervention conditions.
Meija, 2015 [56], Latvia	Case–control unmatched	(31 + 91) (0%) 60.8 ± 6.6 (45–79) ± Prostate cancer (PC)	(A) To investigate the relationship between the intake of bread (particularly rye bread) and the concentration of AR metabolites in urine/plasma in PC and controls and the day and night variation of DHPPA and DHBA (Habitual diet)	3-day food records and 1-day food record (on third day of intervention (analyzed separately)	DHBA, DHPPA Plasma (-) and Urine (12 h and 24 h) HPLC–CEAD	Moderate correlation between U- DHPPA, U-DHBA, and DHPPA plasma (both in 12-h and 24-h urine). Strong to very strong correlation between U-DHBA and U-DHPPA in both in 12-h and 24-h urine. The main exposure variables: bread and bread fiber, rye bread, and rye fiber. 3DFR data were best associated with AR metabolites. Very weak to weak associations between P- and U-metabolites and data from 3 days. Better weak to moderate associations between U-metabolites and the main exposure variables in PC group compared to the controls. Night urine and 24-h urine were best associated with these variables. In PC group, strong correlation between DHPPA plasma and bread and bread fiber, and moderate correlation between rye bread and rye bread fiber (*p* < 0.01)
Knudsen, 2014 [46], European multicenter	Case–control nested	(450 + 450) (46%) Median = 59 (50–64) ± Colorectal cancer	(A) To compare whole-grain intake measured from FFQs and P-AR concentrations. (Habitual diet)	Three different FFQs (every center used a different FFQ)	AR Plasma (pooled F and non-F) GC–MS	Weak correlation between rye, total WG, and P-total-AR (*p* < 0.0001) and inverse correlation with wheat.
Drake, 2014 [60], Sweden	Case–control nested	(1010 + 1817) (0%) 60.8 ± 6.6 (45–73) ± Prostate cancer	(A) To identify major dietary and lifestyle determinants of P-AR metabolites. (Habitual diet)	7-day menu book of lunches and dinners + 168-item dietary questionnaire + 1-h interview (combined)	DHBA, DHPPA and DHBA + DHPPA plasma (non-F) HPLC–CEAD	Weak significant correlations between total fiber, WG, and high bread fiber with DHBA, DHPPA and DHBA + DHPPA (plasma). Very weak significant correlations between total cereal fiber, low-fiber bread with DHBA, DHPPA and DHBA + DHPPA (plasma).
Aubertin-Leheudre, 2008 [34], Finland	Cohort	(56) (100%) 46 ± 13 - Without major diseases ^6^	(A) To examine the relationship between plasma ARs and urinary DHBA and between DHPPA and cereal-fiber intake. Visit 1 in spring Visit 2 in autumn (same year) (Habitual diet)	5-day food records (consecutive days)	AR Plasma (F) and U-DHBA and U-DHPPA (72-h urine) (day-3, -4, -5 FFQ) GC–MS	Significantly weak r total fiber and U-DHBA (not significantly moderate r^13^ with DHPPA). The correlation of cereal fiber was (1) significantly weak with C17:0, C19:0, and C25:0, (2) significantly moderate with C21:0 and C23:0 and total AR, (3) weak with U-DHBA, and (4) moderate with DHPPA. A moderate significant correlation between AR homologs in plasma and U-DHBA and U-DHPPA.
Aubertin-Leheudre, 2010 [59], Finland	Cohort	(56) (100%) 46 ± 13 - Without major diseases ^6^	(A) To evaluate plasma DHBA and DHPPA as biomarkers of whole-grain rye and wheat cereal fiber. Visit 1 in spring Visit 2 in autumn (same year) (Habitual diet)	5-day food records (consecutive days)	P-DHBA and P-DHPPA (F) (day-3, -4, -5 FFQ) HPLC–CEAD	A moderate significant correlation between WGR and total cereal fiber and AR metabolites (DHBA and DHPPA) (plasma) No significant association was detected between plasma AR metabolites and vegetable or berry/fruit fiber intake
Aubertin-Leheudre, 2010 [52], Finland	Cohort	(60) (100%) - - Without major diseases ^6^	(A) To examine the responsiveness of U-AR and P-AR metabolites to rye intake Two time points (V1 and V2) with 6 months later Three groups according to their rye intake: G1 = low rye intake: 23 ± 9 g/day (*n* = 20); G2 = medium rye intake: 44 ± 4 g/day (*n* = 20), G3 = high rye intake: 68 ± 18 g/day (*n* = 20). (Habitual diet)	5-day food records (consecutive days)	P-DHBA, P-DHPPA (F) (day-3, -4. -5 FFQ) U-DHBA, U-DHPPA (day-3, -4, -5 FFQ) HPLC–CEAD	Difference between G1, G2, and G3 was (1) significant in rye and cereal-fiber intake (*p* < 0.05), and (2) non-significant in wheat and total fiber intake (divided groups based on rye intake). Pooled (*n* = 60) r rye intake was (1) moderate with U-DHBA and U-DHPPA (*p* < 0.001), and (2) weak with P-DHBA and P-DHPPA (*p* < 0.05). Weak r between total fiber intake and U-DHBA, U-DHPPA, P-DHBA, P-DHPPA (*p* < 0.05). U-DHBA, U-DHPPA, and P-DHPPA, and (not plasma DHBA) increased proportionally and significantly with the consumption of WGR (good responsiveness).
Linko, 2005 [51], Finland	Cohort	(4+4+1) (-) - - -	(A) To show that whole-grain rye and wheat AR are incorporated into erythrocyte membranes in vivo. I1: No WG 1 week then WG 1 week I2: WG 2 weeks I3: No WG, no gluten 2 weeks (Habitual diet controlled for WG)	4-day diet records (each intervention)	AR Erythrocyte membranes (F) GC–MS	AR homologs are incorporated in the erythrocyte membrane (best for C19:0, C21:0, C23:0). Not detected AR in plasma or erythrocyte membrane in I3 Good symmetric progression in AR in I2 both in plasma and erythrocyte membrane. Unchanged low concentration of AR in I1 both in plasma and erythrocyte membrane.
Ross, 2004 [57], Sweden	Cohort	(1) (0%) 26 26 -	(A) To assess AR metabolites as biomarkers for WGR and WGW intake I1: WG-free diet 5 days I2: High WG single dose (Habitual diet controlled for WG)	Not relevant In-clinic intervention	AR metabolites 12-h urine GC–MS	DHBA and DHPPA were revealed in the urine after consumption of WGR and WGW
Andersson, 2011 [35], Sweden	Cohort	(72) (76%) 42 ± 17 (20–70) Without gastrointestinal diseases	(A) To evaluate (1) the medium-term reproducibility of fasting plasma AR concentrations, (2) the short-term reproducibility of non-fasting plasma AR concentrations, and (3) the relative validity of fasting plasma AR concentrations as an intake biomarker of WG. Visit 1 Visit 2 (after 2–3 months) (Habitual diet)	3-day weighed food records	AR Plasma (F visit 1) and (non-F visit 2) GC–MS	Weak r between P-AR with WGR, total cereal (*p* < 0.05), and moderate with WGW (*p* < 0.001) and (WGR + WGW) (*p* < 0.0001). Strong r between C17.0 and WG rye (*p* < 0.05). Moderate r between WG wheat and C21:0 and C23:0. Positive moderate r between C17/C21 (*p* < 0.0001). Non-fasting P-total-AR was significantly higher than P-total-AR, but the C17:0/C21:0 ratio did not differ between fasting and non-fasting samples. The reproducibility over the period of 2–3 months, when combining the fasting and non-fasting samples was significantly (1) poor for P-total-AR, C25:0, and C23:0, and (2) moderate for C17:0, C19:0, C21:0, and C17:0/C21:0 ratio.
Landberg, 2012 [54], USA	Cohort	(104) (100%) 41.7 ± 3.5 (25–42) Free-living	Long-term reproducibility (1–3 years) and relative validity (r) of U-DHBA and U-DHPPA, and r with WG and cereal fiber Visit 1: Baseline Visit 2: 4 years follow-up Visit 3: 8 years follow-up (A) To evaluate (1) the long-term reproducibility of DHBA and DHPPA in spot urine samples throughout 1–3 years, and (2) the relative validity of the two metabolites as biomarkers of WG, bran, or dietary fiber. (Habitual diet)	151-item semi-quantitative-FFQ	U-DHBA and U-DHPPA (spot urine) GC–MS	Different consumption of WG between occasions. Generally, weak r between U-DHBA, U-DHPPA, and (U-DHBA + U-DHPPA) and (1) WG, cereal fiber and (2) total fiber in V2 and V3. Poor reproducibility of U-DHBA and U-DHPPA (even after adjustment for consumption)
Marklund, 2013 [55], Sweden	Cohort	(66) (76%) 44 ± 17 - Free-living	(A) To evaluate 24-h urinary DHBA and DHPPA as biomarkers by estimating the medium-term (2–3 months) reproducibility and their relative validity compared with self-reported intake of WG, cereal fibers. Visit 1: baseline Visit 2: last day intervention (Habitual diet)	3-day weighted food records -	U-DHBA and U-DHPPA urine (spot and 24 h) GC–MS	The correlation between U-DHBA, DHPPA, and U-(DDHBA + DHPPA) was (1) significantly moderate to strong with WG rye and cereal fibers, (2) significantly moderate with total WG, and (3) non-significantly very weak with WG wheat. (4) statistically non-significant correlation with oat, barley, or rice No difference in WG consumption between 2 occasions. (Poor reproducibility of WG intake) Reproducibility of U-DHBA and U-DHPPA was (1) poor to moderate (ICC = 0.46–0.51) in 24-h urine, and (2) poor in spot urine.
Soderholm, 2009 [61], Finland	Cohort	(15) (53%) 24 ± 5 (20–39) Healthy	(A) To evaluate the short-term reproducibility (hours and up to 1 day) and validity of P-DHBA and P-DHPPA. Baseline: WG-free diet 2 days I1: High WG rye single doses Blood samples collected 3, 4, 5, 6, 7, 8, 10, 12, 14, 16, and 25 h after (Standardized meals)	Not relevant In-clinic intervention	P-DHBA and P-DHPPA (F) HLPC–CEAD	Good reproducibility of DHBA and DHPPA, significantly higher at 25 h than at baseline (*p* < 0.0001) Baseline: P-x-DHBA = 33.2 ± 4.7 and P-x-DHPPA = 35.5 ± 5.9 nmol/L. At 25 h: P-x-DHBA = 103.7 ± 9.5 and P-x-DHPPA = 95.4 ± 10.0 nmol/L nmol/L. P-x-DHBA—tmax = 6.1 ± 0.5 h P-x-DHPPA—tmax = 6.4 ± 0.7 h for DHPPA. P-x-DHBA—t_1/2_ = 10.1 ± 0.8 h P-x-DHPPA—t_1/2_ = 16.3 ± 1.8 h (significantly higher)
Wang, 2017 [63], USA	Cohort	(12) (8%) 35 ± 4 - -	To explore the metabolism and the potential use of avenacosides as a biomarker for WG oat intake. (Habitual diet controlled for cereals)	Not used	Avenacoside metabolites LC–MS	Avenacoside metabolites were absent after Wo and present two hours after a single-dose intake of WG oat. Only a trace of these metabolites was present 36 h after the exposure.
Landberg, 2018 [33], Sweden	Cohort	(40) (50%) 58 ± 5 50–64 Free-living	(A) To identify the reproducibility and the correlation of AR metabolites with WG wheat and rye intake (Habitual diet)	4-day food records (consecutive days)	U-DHBA, U-DHPPA, U-DHCA, U-DHPPTA, U-DHBA-glycine (spot urine day 0, 1, 3, 12, and 14) GC–MS	Poor day-to-day reproducibility. Good reproducibility when analyzing mean day 1 and day 2 vs. mean day 2 and 14 (ICC = 0.75–0.85). No correlation between P-metabolites and U-metabolites (data not reported). The correlation between WG intake and mean (1) DHBA, DHCA, DHBA-glycine was moderate (*p* < 0.05), (2) DHPPA was weak (*p* < 0.05), and (3) DHPPTA was non-significant. The concentration of AR metabolites in urine was highest for DHBA and DHPPA followed by DHCA, DHBA-glycine, and DHPPTA
Wierzbicka, 2017 [32], Sweden	Cohort	(69) (75%) 44 ± 17 - -	(A) To evaluate DHPPTA, DHCA, DHCA-amide, and DHBA-glycine as biomarkers of WGR and WGW intake by assessing their medium-term reproducibility and relative validity. V1: 3DWFR + 24-h urine (day 3) V2: After 2–3 months from V1 3DWFR + 24-h urine (day 3) (Habitual diet)	3-day weighted food records	U-DHBA-glycine, U-DHPPTA, U-DHCA, U-DHCA-amide, U-DHBA, U-DHPPA 24-h urine GC–MS	No significant differences in WG intake between occasions (*p* > 0.05). Poor medium-term reproducibility of WG and AR intake between occasions. The highest urinary excretion reported for DHCA-amide followed DHPPA, DHBA, DHCA, DHBA-glycine, and DHPPTA. DHCA-amide is uniquely an AR derivate. Poor significant reproducibility of P-total AR and its derivates ICC range (0.30–0.39). Moderate reproducibility of U-DHBA-glycine, U-DHPPTA, and U-DHCA, ICC (0.59–0.63). For U-DHPPA and U-DHBA, reproducibility was generally poor. The correlation of WGR and WGW was (1) very weakly insignificant with DHCA-amide and DHBA-glycine, and (2) weak to moderate for other metabolites and total metabolites. Non-significant weak correlation between all metabolites and non-AR-containing cereals (oats, barley and maize)
Zhu, 2014 [58], USA	Cohort	(12) (50%) 1.8 ± 5.5 - Healthy	To explore the metabolism of AR Wo: 3 days At day 4: RG wheat single doses At day 5: WG wheat single doses (Habitual diet low in cereals)	Not relevant In-clinic intervention	U-DHPPTA, U-DHBA-glycine, U-DHBA, and U-DHPPA spot urine (8 time points × 2) Urine (24–32 h) HLPC	The excretion rates of these four metabolites dramatically increased after WG wheat bread consumption, suggesting that all 4 compounds are the metabolites of AR. t_1/2_ (15.9 h for DHBA and 14.8 h for DHPPA) and t-max (8.3 h for 3,5-DHBA and 7.4 h for 3,5-DHPPA) The relative composition of the four metabolites was as follows: U-DHPPTA (3.8%), U-DHBA glycine (6.8%), U-DHBA (24.5%), and U-DHPPA (65.0%). (DHBA, DHPPTA still the major components of AR)
Wu, 2018 [47], Sweden	Cohort	(258) (42%) - - Free-living	(A) To evaluate AR in adipose tissue biopsies as a biomarker of long-term WGR and WGW intake Biopsies in (2003–2009 women and 2010–ongoing for women) For men, correlation between P-AR and WG intake last two years (FFQ 2009–2010) and 14 years (FFQ 1997–2003). For women, 7 years (1997–2003) and 17 years (FFQ 1987–1997) (Habitual diet)	Self-administered semi-quantitative FFQ (at three different endpoints during 14–17 years) (analyzed separately)	AR Plasma (F) and adipose tissue GC–MS	In data from last FFQ (few years before biopsies), weakly significant r_s_ between WGR and WGR + WGW and all AR homologs, except moderate r_s_ for WGR and C17:0. Very weak correlation between WGW and all homologs. Generally weakly significant correlations between WG intake and P and A-AR in the long-term assessment. The correlation between plasma and adipose AR is very strong in C17:0, strong in C19:0 and C21:0, moderate in C23:0 and C25:0 (*p* < 0.001)
Landberg, 2011 [48], Denmark	Cross-sectional	(360) (100%) 56 (53–60) Free-living	(A) To estimate the variation in plasma AR concentration (Habitual diet)	192-item FFQ	AR Plasma (non-F) GC–MS	r P-AR and all homologs are (1) weakly significant with Rye bread and (2) very weakly significant with cereal fibers and total fibers
Guyman, 2008 [53], USA	Cross-sectional	(99) (47%) - (20–39) Healthy and non-smoking	(A) To determine the utility of DHPPA as a biomarker for WG intake by investigating the relationship between whole-grain wheat and rye intake and DHPPA excretion from 3-day food records and 12-h urine at day 4. (Habitual diet)	3-day food records (consecutive days) and FFQ (analyzed separately)	U-DHPPA 12-h overnight urine LC–MS	From both 3DFR and FFQ data, WGR + WGW intake and WG intake was associated with DHPPA excretion. From 3DFR data, the DHPPA excretion in WGR + WGW consumers was 44% higher than no-consumers (ratio of excretion (95% CI) 1.44 (1.04, 1.97); *p* = 0.029) (adjusted for BMI, energy, and fiber) From FFQ data: (1) A serving increase in WG intake increased DHPPA by 67% (2) A serving increase in whole-grain wheat 1 rye intake increased DHPPA excretion by 94%
McKeown, 2016 [49], USA	Cross-sectional	(190) (100%) 65 (SE = 0.5) - Coronary disease	(A) To investigate the association between plasma AR concentrations and estimates of dietary intake derived from self-reported FFQ (Habitual diet)	226-item FFQ	AR Plasma (-) GC–MS	Weak significant r between P-total-AR and WG, total fiber, cereal fiber, and very weak with legume fiber. Non-significant weak correlations with RG, fruit, and vegetable fibers.
Jansson, 2010 [50], Sweden	Cross-sectional	(20) (100%) - - Free-living	(A) To investigate AR content and relative homologue composition in adipose tissue biopsies Assessment of AR as a long-term biomarker (Habitual diet)	123-item FFQ	AR Plasma (F) and adipose tissue GC–MS	Moderate significant r between WG bread and total AR adipose tissue (r 0.48, *p* < 0.05)

Note: ^1^ The total number of the participants included in analyses. ^2^ The proportion of women as a percentage. ^3^ Values are presented as means ± SD and range. ^4^ Reported as subjects without diseases or conditions like a strong mental handicap, severe nutrient malabsorption, and strong food intolerances or allergies, concomitant participation in other scientific studies that involved radiation or blood sampling. ^5^ Reported as healthy subjects with at least one self-reported gastrointestinal symptom (such as flatulence, bloating, abdominal pain, constipation, or diarrhea) after consumption of cereal foods, especially rye bread. ^6^ Reported as subjects with no history of cancer or other major diseases or using oral contraceptives, hormone replacement therapy, or antibiotics. Abbreviations: (-), not reported; 3DFR, 3-day food records; 3DWFR, 3-day weighted food records; A, the aim; AR, alkylresorcinol; CI, confidence interval of the mean; BMI, body mass index; DHBA, 3,5-dihydroxybenzoic acid; DHBA-glycine, 2-(3,5-dihydroxybenzamido)acetic acid; DHCA, (3,5-dihydroxycinnamic) acid; DHCA-amide, 3,5-dihydroxycinnamic acid amide; DHPPA, 3-(3,5-dihydroxyphenyl)-1-propanoic Acid; DHPPTA, 5-(3,5-dihydroxyphenyl)pentanoic acid; F, fasting samples; FFQ, food frequency questionnaire; G, group; GC–MS, gas chromatography–mass spectrometry; HPAA, N-(2-hydroxyphenyl) acetamide; HHPAA, hydroxy-N-(2-hydroxyphenyl) acetamide; I, intervention; LC–QTOF-MS, liquid chromatography–quadrupole time-of-flight mass spectrometry; LC–MS, liquid chromatography–mass spectrometry; non-F, non-fasting samples; P, plasma; RCT, randomized controlled trial; RF, refined grains; U, urine; UPHLC, Ultra-high-performance liquid chromatography; HPLC–CEAD, high-performance liquid chromatography with coulometric electrode array detection; SE, standard error; UK, United Kingdom; USA, United States of America; V, visit or time point; WG, whole grains; WGR, whole-grain rye; WGR + WGW, whole-grain rye and wheat; WGW, whole-grain wheat; Wo, washout period; x, the mean of the concentration.

**Table 3:** The main characteristics of the included studies applying a non-targeted metabolomic approach.

Study (Author, Year, Country)	Study Design	Population (*n*) ^1^ (% ♀) ^2^ Age (years) ^3^ Health Status	Aim(A), Intervention(I), Description (Background Diet)	Method of the Report of the Exposure	Biological Sample
Bondia-Pons, 2013 [64], Finland	RCT cross-over, non-blinded	(20) (50%) ♀: 40.6 ± 7.7 ♂: 43.4 ± 9.9 ^4^ - hypercholesterolemia	(A) To elucidate urinary biomarkers of WGR intake by a non-targeted UPLC–QTOF-MS metabolite profiling I1: Rye bread 4 weeks Wo: 4 weeks I2: Wheat bread 4 weeks (Habitual diet controlled for WG bread products)	4-day food records	24-h urine
Johansson-Persson, 2013 [65], Sweden	RCT cross-over, non-blinded	(25) (60%) - (49–66) Overweight healthy	(A) To investigate the alteration in the plasma metabolome profile in high dietary fiber diet by non-targeted LC–QTOF-MS I1: High fiber (x = 48.0 g) 5 weeks Wo: (-) 3 weeks I2: Low fiber (x = 32.2 g) 5 weeks (Habitual diet controlled for fiber)	3-day food records (consecutive days) and daily FFQ	Plasma (F)
Hanhineva, 2015 [66], Finland	RCT parallel, non-blinded	(106) (-) - 40–70 impaired glucose concentration in the blood	(A) To report novel biomarkers for the consumption of WG, bilberries, and fish by a non-targeted LC–MS I1: Healthy diet containing WG, fatty fish, and bilberries (*n* = 37) I2: WG-enriched diets, habitual eating of fish and berries (*n* = 34) I3: Control diet with refined wheat bread, no fish and berries (*n* = 35) (Controlled feeding trail)	4-day dietary records	Plasma (F)
Zhu, 2016 [67], USA	Cohort	(12) (50%) 1.8 ± 5.5 - Healthy	(A) To analyze metabolites from WGW bread and RF wheat bread intake using (1) non-targeted UPLC–MS/MS (2) targeted HPLC–MS/MS metabolomics Wo: 3 days At day 4: RF wheat single dose At day 5: WGW single dose (Habitual diet low in cereals)	Not used In-clinic intervention	24-h urine at six time points on day 4 and 5
Coulomb, 2015 [68], Sweden	Cohort	(1) (0%) 35 Healthy	(A) To search for the discriminative metabolites in the endosperm and bran of WGR and WGW by non-targeted NMR-based metabolomics Refined wheat bread six days On day seven WGR bread (Habitual diet controlled for WG)	Not used	24-h urine at day 6 and 7
Garcia-Aloy, 2014 [69], Spain	Cross-sectional	(155) (-) - 55–80 Type-2 diabetes/cardiovascular risk factors	(A) To elucidate biomarkers of bread exposure by non-targeted HPLC–QTOF-MS. Non-consumers of bread (*n* = 56) White-bread consumers (*n* = 48) WG-bread consumers (*n* = 51) (Habitual diet)	137-item FFQ	Spot urine
Hanhineva, 2015 [70], Sweden	Cross-sectional	(66) (75%) 44 ± 17 - Free-living	(A) (1) To discover putative biomarkers for WGR intake by non-targeted LC–MS (2) To identify the reproducibility of identified markers in samples taken 1–3 months apart (Habitual diet)	3-day weighted food records	24-h urine

Note: ^1^ The total number of the participants included in analyses. ^2^ The proportion of women as a percentage. ^3^ Values are presented as means ± SD and range. ^4^ The mean of the age is presented separately for men and women. Abbreviations: (-), not reported; A, the aim; F, fasting samples; UPLC–QTOF-MS, ultra-performance liquid chromatography–quadrupole time-of-flight mass spectrometry; FFQ, food frequency questionnaire; LC–QTOF-MS, liquid chromatography–quadrupole time-of-flight mass spectrometry; LC–MS, liquid chromatography–mass spectrometry; I, intervention; UPLC–MS/MS, ultra-performance liquid chromatography–tandem mass spectrometry; HPLC–MS/MS, high-performance liquid chromatography–tandem mass spectrometry; P, plasma; RCT, randomized controlled trial; RF, refined grains; U, urine; WG, whole grains; WGR, whole-grain rye; NMR, nuclear magnetic resonance; HPLC–QTOF-MS, high-performance liquid chromatography–quadrupole time-of-flight mass spectrometry; WGW, whole-grain wheat; Wo, washout period; x, the mean concentration.

**Table 4:** The reported metabolites and main results of the included studies applying a non-targeted metabolomic approach.

Reported Metabolites	Bondia-Pons 2013 [64]	Johansson-Persson 2013 [65]	Hanhineva 2015 [66]	Zhu 2016 [67]	Coulomb 2015 [68]	Garcia-Aloy 2014 [69]	Hanhineva 2015 [70]
	Biological Sample						
	Urine	Plasma	Plasma	Urine	Urine	Urine	Urine
2,6-DHBA		X^2^					
2,8-Dihydroxyquinoline glucuronide						X^8^	
2-Aminophenol sulfate	X^1^	X^2^		X^4^		X^7^	
3,5-DHPPA glucuronide	X^1^					X^8^	
3,5- DHPPA sulfate	X^1^			X^4^			S^9^ (*r* = 0.61; *p* < 0.001)
3,5- DHPPTA sulfate				X^4^			
3,5-DHBA				X^4,5^			
3,5-DHBA glycine				X^4^			
3,5-DHBA sulfate				X^4^			
3,5-DHPHTA sulfate				X^4^			
3,5-DHPPA derivative (fragmented ion)							S^9^ (*r* = 0.64; *p* < 0.001)
3,5-DHPPTA				X^4^			
3,5-Dihydroxyhydrocinamic acid sulfate	X^1^						
3,5-Dihydroxyphenyl ethanol sulfate	X^1^						
3-Indolecarboxylic acid glucuronide						X^8^	
3-Methylcatechol sulfate				X^5^			
Alkenylresorcinol 21:1-Gln			S^3^ (*r*_s_ = 0.63; *p* < 0.05)				
Alkylresorcinol 19:0 Gln			M^3^ (*r*_s_ = 0.47; *p* < 0.05)				
Azelaic acid (nonanedioic acid)	X^1^				X^6^		
Caffeic acid sulfate				X^4^			M^9^ (*r* = 0.58; *p* < 0.001)
DIBOA sulfate	X^1^						
Dihydroferulic acid sulfate						X^8^	
Enterolactone glucuronide	X^1^					X^8^	
Ferulic acid-4-*O*-sulfate	X^1^			X^4,5^			
Feruloyglycine				X^4^			
Feruloyglycine sulfate				X^4^			
HBOA glycoside						X^8^	
HHPAA						X^8^	M^9^ (*r* = 0.54; *p* < 0.001)
HHPAA sulfate				X^4^			S^9^ (*r* = 0.62; *p* < 0.001)
HHPPA sulfate				X^4^			
HMBOA						X^7^	
HMBOA glucuronide						X^7^	
HPAA glucuronide						X^7^	
HPAA sulfate				X^4,5^			M^9^ (*r* = 0.54; *p* < 0.001)
HPPA				X^4^		X^7^	
Hydroxybenzoic acid glucuronide						X^7^	
Indolylacryloylglycine	X^1^						
Pimelic acid							M^9^ (*r* = 0.58; *p* < 0.001)
Pyrraline						X^8^	
Riboflavin						X^8^	

Note: ^1^ Increase in urine from participants eating WGR bread compared to refined wheat bread. ^2^ Increase in fasting plasma after eating high-fiber diet containing oat bran, rye bran, and sugar beet fiber compared to low-fiber diet. ^3^ Correlated with whole-grain bread consumption. ^4^ Significant fold increase two to six hours after consumption of WG wheat compared to RF wheat identified using a targeted approach. ^5^ Significant fold increase two to six hours after consumption of WG wheat compared to RF wheat, identified using a non-targeted approach. ^6^ Increased at 24 h after consumption of WG rye bread. ^7^ Increased in participants eating whole-grain bread compared to non-consumers of bread; however, no difference was observed between whole-grain-bread and white-bread consumers. ^8^ Increased in whole-grain-bread consumers compared to both no-bread and white-bread consumers. ^9^ Reported correlation with rye consumption. Abbreviation: X, identified; S, strong; M, moderate; WGR, whole-grain rye; RF, refined grains; WG, whole grains.

## Supplementary Materials

The following are available online at https://www.mdpi.com/2072-6643/11/12/2994/s1: Table S1: The search strategy used in different databases; Table S2. Quality assessment and the assessment of the risk of bias in the included studies

## Abbreviations

WG	whole grain
FFQ	food frequency questionnaire
CI	95% confidence interval
SD	standard deviation
SE	standard error
ICC	intraclass correlation coefficient
AR	alkylresorcinol
P-AR	plasma alkylresorcinol
WGR	whole-grain rye
WGW	whole-grain wheat
3DFDs	3-day food diaries
DRs	daily records
h	hours
RF	refined grain
EM	erythrocyte membrane
DHBA	3,5-dihydroxybenozoic acid
DHPPA	3-(3,5-dihydroxyphenyl)-1-propanoic acid
DHCA	3,5-dihydroxycinnamic acid
DHBA-glycine	2-(3,5-dihydroxybenzamido)acetic acid
DHPPTA	5-(3,5-dihydroxyphenyl)pentanoic acid
DHCA-amide	3,5dihydroxycinnamic acid amide
DIBOA	2,4-dihyxdoxy 1,4-benzoxazin-3one
HHPAA	hydroxy-*N*-(2-hydroxyphenyl) acetamide
HPAA	*N*-(2hydroxyphenyl) acetamide
