# Supplementary material for: Biomarkers of Whole-Grain and Cereal-Fiber Intake in Human Studies: A Systematic Review of the Available Evidence and Perspectives
Source: Nutrients. 2019 Dec 6;11(12):2994. doi: 10.3390/nu11122994 (PMC6950731; doi:10.3390/nu11122994)
Supplement: Supplementary file 1 [file nutrients-11-02994-s001.pdf]

Table S1. The search strategy used in different databases.

**Medline via OVID**	#1 exp dietary fiber/<br>#2 dietary fiber*.tw<br>#3 dietary fibre*.tw<br>#4 Roughage*.tw<br>#5 wheat bran*.tw<br>#6. whole grain*.tw<br>#7 alimentary fiber*.tw<br>#8 alimentary fibre*.tw<br>#9 biomarker*.tw<br>#10 biologic marker*.tw<br>#11 bioindicator*.tw<br>#12 biological marker*.tw<br>#13 exp biological marker/<br>#14 biological indicator*.tw<br>#15 1 or 2 or 3 or 4 or 6 or 7 or 8<br>#16 9 or 10 or 11 or 12 or 13 or 14<br>#17 15 and 16<br>#18 17 not (exp animals/ not humans.sh.)
**EMBASE via OVID**	#1 exp dietary fiber/<br>#2 dietary fiber*.tw<br>#3 dietary fibre*.tw<br>#4 Roughage*.tw<br>#5 wheat bran*.tw<br>#6. whole grain*.tw<br>#7 alimentary fiber*.tw<br>#8 alimentary fibre*.tw<br>#9 biomarker*.tw<br>#10 biologic marker*.tw<br>#11 bioindicator*.tw<br>#12 biological marker*.tw<br>#13 exp biological marker/<br>#14 biological indicator*.tw<br>#15 1 or 2 or 3 or 4 or 6 or 7 or 8<br>#16 9 or 10 or 11 or 12 or 13 or 14<br>#17 15 and 16<br>#18 17 not ((exp animal/ or nonhuman/) not exp human/)
**Web of Science**	# 1 TS = ("dietary fiber*" or "dietary fibre*" or roughage* or "whole grain*" or "alimentary fiber*" or "alimentary fibre*" or "wheat bran*")<br>#2 TI = ("Biological marker*" or "Biomarker*" or "Biologic marker*" or "Biological indicator*" or Bioindicator* or "biologic marker*")<br>#3 #2 AND #1
**The Cochrane database**	#1 "dietary fiber*":ti,ab,kw<br>#2 "dietary fibre":ti,ab,kw<br>#3 "roughage":ti,ab,kw<br>#4 "wheat bran":ti,ab,kw<br>#5 "whole grain":ti,ab,kw<br>#6 alimentary fiber*:ti,ab,kw<br>#7 alimentary fibre:ti,ab,kw<br>#8 MeSH descriptor: [Dietary Fiber] explode all trees<br>#9 #1 or #2 or #3 or #4 or #5 or #6 or #7 or #8<br>#10 MeSH descriptor: [Biomarkers] this term only

	#11 biomarker*:ti,ab,kw<br>#12 biologic marker*:ti,ab,kw<br>#13 bioindicator*:ti,ab,kw<br>#14 biological marker*:ti,ab,kw<br>#15 biological indicator*:ti,ab,kw<br>#16 #10 or #11 or #12 or #13 or #14 or #15<br>#17 #9 and #16

Table 2. Quality assessment and the assessment of the risk of bias in the included studies.

Study			Methods			Assessment		Analyses							
**Author**	**Study design**	**Total Sample size**	**Reported inclusion and exclusion criteria**	**Reported health status**	**Well-described intervention/set-up**	**Qualified assessor**	**Blinded assessor**	**Adjusted for potential confounders**	**Same apparatus (calibration) for all participants**	**Valid questionnaire for the study**	**Recall period for grain intake in questionnaire**	**Time period assessed by questionnaire**	**Blood samples within 24 h since last intervention day?**	**Fasting blood samples?**	**Type of urine collection**
Ampatzoglou, 2015 [36]	RCT X-over non-blinded	33	Y	Y	Y	Y	-	N	Y	-	<3 d	6 w	Y	Y	#
Biltoft-Jensen, 2016 [37]	RCT X-over non-blinded	750	Y	Y	Y	Y	-	Y	Y	-	<3 d	3 m	N	Y	#
Landberg, 2008 [38]	RCT X-over non-blinded	30	N	N	Y	-	-	Y	Y	-	1 d	6 w	Y	Y	#
Landberg, 2009 [39]	RCT X-over n/blinded	16	N	Y	Y	-	N	Y	Y	-	7 d	1 d	Y	Y	24 h
Linko, 2005 [40]	RCT X-over non-blinded	39	Y	Y	Y	-	-	N	-	-	-	8 w	-	-	#
Hanhineva, 2014 [62]	RCT X-over non-blinded	12	Y	Y	N	Y	-	N	Y	#	#	#	Y	Y	#
Magnusdottir, 2013 [41]	RCT parallel non-blinded	158	Y	Y	Y	Y	-	Y	N	-	1 d	18-24 w	-	-	#
McKeown, 2016 [42]	RCT X-over non-blinded	19	Y	Y	Y	-	N	Y	-	-	3 d	6 d	Y	Y	24 h
Wu, 2015 [43]	RCT parallel non-blinded	16	Y	Y	Y	-	-	N	Y	-	1 d	<4 w	-	Y	#
Landberg, 2009 [44]	RCT X-over non-blinded	17	Y	Y	Y	Y	N	Y	Y	-	1 d	6 w	#	Y	#
Ross, 2012 [45]	RCT parallel non-blinded	266	Y	Y	Y	Y	N	Y	-	Y	1 d	16 w	-	Y	#
Knudsen, 2014 [46]	Case–Control Nested	900	Y	Y	Y	-	-	Y	Y	Y	-	-	-	Y/N	#
Drake, 2014 [60]	Case–Control Nested	2927	Y	Y	N	Y	-	Y	Y	-	<1 d	1 d	-	N	#

Meija, 2015 [56]	Case–Control Unmatched	31	Y	Y	Y	Y	-	N	Y	-	<1 d	1 d	Y	Y	12 h
Aubertin-Leheudre, 2008 and 2010 [34,59]	Cohort	56	Y	Y	Y	Y	-	Y	Y	Y	1 d	1 d	Y	Y	72 h
Aubertin-Leheudre, 2010 [52]	Cohort	60	Y	Y	Y	Y	-	Y	Y	Y	1 d	1 d	Y	Y	72 h
Andersson, 2011 [35]	Cohort	72	Y	Y	Y	-	-	N	-	-	1 d	1 d	N	Y/N	#
Linko, 2005 [51]	Cohort	9	N	N	N	N	-	N	-	/	-	1-2 w	y	Y	#
Landberg, 2012 [54]	Cohort	104	Y	N	Y	Y	N	Y	-	-	1 yr	1 yr	#	#	spot
Marklund, 2013 [55]	Cohort	66	Y	N	Y	Y	-	Y	Y	-	<1 d	<1 d	#	#	24 h
Soderholm, 2009 [61]	Cohort	15	Y	Y	Y	-	-	N	Y	#	#	#	Y	Y	#
Ross, 2004 [57]	Cohort	1	N	N	N	Y	-	#	N	Y	#	#	#	#	12 h
Landberg, 2018 [33]	Cohort	40	Y	N	Y	Y	-	Y	Y	Y	<1 d	<1 d	#	#	spot
Wierzbicka, 2017 [32]	Cohort	69	Y	N	Y	-	-	N	-	Y	<1 d	<1 d	Y	-	24 h
Wang, 2017 [63]	Cohort	12	Y	Y	Y	-	-	#	Y	#	#	#	#	#	48 h at 8 intervals
Zhu, 2014 [58]	Cohort	12	Y	Y	Y	-	-	N	Y	#	#	#	#	#	24–36 h
Wu, 2018 [47]	Cohort	258	Y	N	Y	-	-	Y	-	-	1 yr	1 yr	N	Y	#
Landberg, 2011 [48]	Cross-sectional	360	N	N	N	-	-	Y	Y	-	1 yr	1 yr	Y	N	#

Guyman, 2008 [53]	Cross-sectional	99	Y	Y	Y	Y	-	Y	Y	-	<3 m	<3 m	#	#	12 h
McKeown, 2016 [49]	Cross-sectional	190	Y	Y	Y	-	-	Y	Y	-	< 1 d	1 yr	-	-	#
Jansson, 2010 [50]	Cross-sectional	20	N	N	N	-	N	N	Y	-	1 yr	1 yr	-	F*	#
Bondia-Pons, 2013 [64]	Untargeted metabolomic RCT- X-over n/blinded	20	Y	Y	Y	Y	-	-	Y	-	1 d	4 w	#	#	24 h
Johansson-Persson, 2013 [65]	Untargeted metabolomic RCT-X-over n/blinded	25	Y	Y	Y	-	Y	N	Y	-	1 d	5 w	Y	Y	#
Hanhineva, 2015 [66]	Untargeted metabolomic RCT- Parallel n/blinded	106	Y	Y	Y	-	-	-	Y	-	1 d	3 w	y	y	#
Zhu, 2016 [67]	Untargeted metabolomic Cohort	12	Y	Y	Y	-	-	N	Y	#	#	#	#	#	2 × 24-h urine at 6 time points
Coulomb, 2015 [68]	Untargeted metabolomic Cohort	1	N	N	Y	-	-	N	Y	#	#	#	#	#	24 h
Garcia-Aloy, 2014 [69]	Untargeted metabolomic Cross-sectional	155	Y	N	Y	-	-	N	Y	Y	1 m	1 m	#	#	Spot urine
Hanhineva, 2015 [70]	Untargeted metabolomic Cross-sectional	66			Y	-	-	N	Y	-	1 d	1 d	#	#	24 h

Note: Abbreviation: (-), not reported; (#), not applicable; d, day; h, hours; RCT, randomized controlled trial; x-over, cross over design; N, no; w, week; Y, yes; yr, year;
